# Seeing the Meaning: Top–Down Effects on Letter Identification

**DOI:** 10.3389/fpsyg.2017.00322

**Published:** 2017-04-20

**Authors:** Gemma A. L. Evans, Matthew A. Lambon Ralph, Anna M. Woollams

**Affiliations:** Neuroscience and Aphasia Research Unit, School of Psychological Sciences, University of ManchesterManchester, England

**Keywords:** letter identification, Reicher–Wheeler, word superiority, semantics, imageability, semantic priming

## Abstract

Most models of reading agree that visual word recognition is underpinned by a highly interactive network in which both bottom–up and top–down processes contribute. What remains unknown is whether evidence of top–down effects upon letter processing are restricted to word-form level information, or whether meaning-level information also plays a role. Here we sought to investigate top–down semantic influences upon letter detection using semantic manipulations of real word imageability and semantic priming, as well as a manipulation of nonword orthographic and phonological composition which varied degree of similarity to real words. A continuous adaptive staircase procedure was used, allowing us to assess the exposure duration needed for accurate letter perception in different stimulus types. Results revealed that in terms of both exposure duration and decision reaction times, words showed an advantage over pseudohomophones and pseudowords, which in turn showed advantages over consonant strings. High imageability words were processed more efficiently than low imageability words, both in terms of the exposure duration required for accurate letter identification and also decision reaction times. The presence of a related as opposed to unrelated semantic prime significantly shortened exposure duration, but also lengthened decision reaction times. This inhibitory semantic priming effect in reaction time was attributed to the interference at the decision stage by stronger activation of the prime letters in the case of related relative to unrelated trials. Taken together, the present results establish for the first time that the semantic dimensions of imageability and semantic priming exert significant effects on letter identification, indicating meaning-level influences on the very earliest stages of written word recognition.

## Introduction

Within a few hundred milliseconds of encountering a written word we are able to ascertain one of countless intended meanings from only minor variations in a finite number of alphabetic symbols. Given the speed and efficiency of normal word recognition in the face of these high perceptual demands, it seems unlikely that word processing is underpinned solely by a strict hierarchal bottom-up analysis of visual input, as early reading models originally suggested (e.g., Forster, 1976). Indeed both localist and connectionist reading models now incorporate a commitment to cascaded and interactive processing, whereby partial activation of representations may pass freely between different levels of representation throughout the course of word processing (Rumelhart and McClelland, 1982; Grainger and Jacobs, 1994; Coltheart et al., 2001; Harm and Seidenberg, 2004; Perry et al., 2007). This interactivity enables both bottom–up and top–down processes to simultaneously and iteratively contribute to word recognition. There is clear evidence that higher-level knowledge can contribute to letter identification, as demonstrated by observations that letters are more accurately identified when processed within the context of real words relative to when they are processed in isolation (Reicher, 1969; Wheeler, 1970; Rumelhart and McClelland, 1982). What remains unclear, however, is the extent to which this higher-level knowledge is meaning-based.

Within localist models (Coltheart et al., 2001; Perry et al., 2007), words are represented as lexical nodes within an orthographic input lexicon. Activation from the semantic system may feed back to this level, producing meaning-level effects on word recognition, and activation from the orthographic input lexicon can in turn feed back to the letter units to influence letter processing. Within distributed connectionist models (Seidenberg and McClelland, 1989; Plaut et al., 1996; Harm and Seidenberg, 2004) lexical knowledge is represented as distributed patterns of activity across the three interacting systems of orthography, phonology, and semantics, hence the semantic system can similarly feed back to influence letter processing. Both localist and connectionist models therefore are compatible with the proposal that semantic information can influence letter processing via feedback.

Evidence that lexical information can influence letter processing has been derived from investigations exploring the effect of context upon letter identification. This has been typically investigated using the Reicher-Wheeler paradigm (Reicher, 1969; Wheeler, 1970) in which participants are visually presented with a masked letter string under brief exposure durations and required to identify which of two alternative letters was present in the display. In addition to an identification advantage for letters presented in real word contexts relative to those in isolation, this paradigm has also been used to elicit an identification advantage for letters presented in the context of real words relative to letters presented in the context of nonwords (e.g., *D* is perceived more accurately when presented in *DOG* relative to *DEG*; Reicher, 1969; Wheeler, 1970; Rumelhart and McClelland, 1982); a phenomenon termed the word superiority effect.

Context effects upon letter processing also extend to nonwords, with increasingly word-like nonword compositions being found to produce increasingly accurate letter identification. The pseudohomophone superiority effect reflects the finding that letters are more accurately identified when presented in the context of pseudohomophones relative to pseudowords (e.g., *P* is perceived more accurately in *PAED* than *PALD*; Chastain, 1987). The pseudoword superiority effect reflects the finding that letters are more accurately identified when presented in the context of orthographically legal pseudowords, relative to illegal consonant strings (e.g., *F* is more accurately perceived when presented in the context of *FLUP* relative to *FLRP*; Grainger et al., 2003). Finally, letters have also been found to be more accurately identified when presented in the context of illegal consonant strings that share a high degree of letter overlap with real words relative to illegal consonant strings that do not share such overlap (e.g., *S* is more accurately perceived in the context of *SLNT* relative to *XLQJ*; Rumelhart and McClelland, 1982). Collectively these words and word-like nonword advantages have been widely interpreted as demonstrating top–down lexical influences upon letter-level processing (Rumelhart and McClelland, 1982; cf. Grainger and Jacobs, 2005), however whether these effects are accompanied by semantic-level feedback remains undetermined.

Our previous work has provided evidence for a functional role for semantic information in visual word recognition using the lexical decision task (Evans et al., 2012). In that study, we manipulated both intra- and an inter-word semantic variables: imageability and semantic priming, respectively. Imageability refers to the ease with which a word’s meaning invokes a mental image, as measured by subjective ratings (Paivio et al., 1968). In models of semantic representation, words with high imageability meaning such as *HARP* enjoy a processing advantage over low imageability words like *HOPE* by virtue of having a greater number of semantic features (Plaut and Shallice, 1993) and a higher degree of intercorrelation between their features (Harm and Seidenberg, 2004). Semantic priming refers to the performance benefit associated with presentation of a prime word related in meaning before a target (e.g., *cat–*DOG) relative to presentation of an unrelated prime word (e.g., *cup*–DOG). Connectionist models of semantic representation have simulated semantic priming effects in lexical decision in terms of the related primes and targets sharing a greater number of semantic features and more often co-occurring in language than unrelated primes and targets (Plaut and Booth, 2000).

In lexical decision, the difficulty of the discrimination is crucially determined by the nature of the nonword foils. Evans et al. (2012) manipulated the wordlikeness of the foils, from pseudohomophones orthographically matched to the words (e.g., BRANE), through pseudowords orthographically matched to the words (e.g., BRONE), to consonant strings (e.g., BRXNE). Both imageability and semantic priming effects varied parametrically with foil type, such that they were largest with the pseudohomophone foils, significantly smaller but still reliable with the pseudoword foils, and absent with the consonant string foils. These results support the central role attributed to semantic information in connectionist models when lexical decision cannot be made on the basis of orthographic or phonological form, which is a consequence of their reliance on distributed orthographic representations (Seidenberg and McClelland, 1990; Plaut, 1997). In models where lexical decision is based on orthographic information (e.g., Harm and Seidenberg, 2004), the observed pattern of semantic effects could have arisen due to feedback from the semantic level. Alternatively, however, the observed sematic effects could have arisen as a consequence of differential weighting of semantic activation in a global decision metric, and indeed such an approach has successfully simulated our data concerning the impact of foil type on the size of the imageability effect (Chang et al., 2013).

Letter identification is a task in which response can be driven purely by activation at the orthographic level, and no semantic information is required. The presence of semantic effects in letter identification would therefore provide evidence supporting a pervasive influence of semantic feedback. The goal of the present study was therefore to explore whether top–down semantic information can influence letter identification performance in the same way as previously proposed for feedback from the lexical level. Building upon our previous work in lexical decision (Evans et al., 2012), lexical influences upon letter processing were examined in the present study by using the nonword foils that varied in their orthographic and phonological composition in order to parametrically manipulate their similarity to real words. Hence letter detection was assessed within real word (e.g., *BRAIN*), pseudohomophone (e.g., *BRANE*), pseudoword (e.g., *BRANT*) and consonant string items (e.g., *BRPNT*). Semantic influences upon letter processing were examined by using the word targets that varied either in imageability of the word’s referent (e.g, *HARP vs. HOPE)* or semantic relatedness of a preceding prime word (e.g., *cat–DOG vs. cup–DOG)*.

Letter processing was examined using a modified Reicher–Wheeler paradigm (Reicher, 1969; Wheeler, 1970) in which participants viewed briefly presented masked words before performing a two-alternative forced-choice letter identification task. Studies using this technique have generally adopted a fixed display duration brief enough to bring performance sufficiently below ceiling to allow examination of the impact of different manipulation on accuracy. Given evidence from neuroimaging for rapid activation of semantic information in visual word recognition (Hauk et al., 2006; Sysoeva et al., 2007; Wirth et al., 2008), we were particularly interested in examining the very early stages of orthographic processing. To achieve this, rather than fix display duration and examine accuracy, we chose to fix accuracy by means of a continuous adaptive staircase procedure and examine display duration as a dependent variable. When combined with blocked stimulus presentation, allowed us to quantify the exposure durations needed for accurate perception of letters in different kinds of strings. We also considered reaction time, a measure not traditionally considered in studies employing the Reicher–Wheeler paradigm, as we expected this to provide an index not only of letter perception but also of decision processes.

On the basis of previous research, we expected to see a word superiority effect, such that exposure durations and reaction times would be significantly shorter for words relative to all nonwords. The differences amongst the nonword conditions would be expected to inversely correspond to those seen when these items were used as lexical decision foils, because increasing wordlikeness is disadvantageous in lexical decision but advantageous in perceptual identification. A pseudohomophone superiority effect would take the form of shorter exposure durations and reaction times for pseudohomophones than the other nonwords. A pseudoword superiority effect was expected in the form of shorter exposure durations and reaction times for pseudowords than consonant strings. Turning to semantic effects, we expected these to correspond to those seen with these words in lexical decision. Hence exposure durations and reaction times should be shorter for high than low imageability words and for words preceded by a semantically related versus unrelated prime. To the extent that the phonology of the pseudohomophones activated the semantics of their baseword, effects of imageability and semantic priming could also be expected for these items. In summary, we expected that effects of lexicality, homophony and legality would be accompanied an influence of meaning-level variables upon letter processing.

## Materials and Methods

### Participants

Sixty-four undergraduate students completed both the imageability and semantic priming letter detection tasks. Task order was counterbalanced between participants. Participants reported that they had normal or corrected-to-normal vision, no identified reading disorders and English was their first language. The research was approved by the University of Manchester School of Psychological Sciences Research Ethics Committee.

### Stimuli

#### Imageability

Each participant was presented with 80 real words and 240 nonwords. The real words were monosyllabic, 3–5 letter, low-frequency English words. Imageability ratings taken from the Cortese and Fugett (2004) database were used to select 40 low imageability (e.g., *OWE*) and 40 high imageability (e.g., *FUR)* words, which were presented in two separate blocks.

Three types of nonwords were created: pseudohomophones, pseudowords and consonant strings. Eighty orthographically legal pseudohomophones were created, 40 of which had low imageability basewords (e.g., *NUM*) and 40 of which had high imageability basewords (e.g., *BEA)*, which were again presented in two separate blocks. For each pseudohomophone a letter was replaced to create 80 orthographically legal, pronounceable pseudoword foils (e.g., *JUM*), and vowels were replaced with consonants to create 80 orthographically illegal, unpronounceable consonant strings (e.g., *JKM*). Again, the pseudowords and consonant strings were presented in two blocks of 40 items according to the pseudohomophone from which they were derived.

In accordance with the standard Reicher-Wheeler paradigm (Reicher, 1969; Wheeler, 1970), both a correct target and an incorrect foil letter were presented following each item, one placed above and one below a particular position in the pattern mask. The incorrect foil letter was selected such that if it replaced the correct letter at the position probed it would make an alternative real word for real word items (e.g., *OWE probed with O or A*), an alternative real word for pseudohomophone items (e.g., *BEA probed with A and T*), an alternative orthographically legal pseudoword for pseudoword items (e.g., *JUM probed with J and L*) and an alternative consonant string for consonant string items (e.g., *JKM probed with J and P*). This criterion was implemented to reduce the influence of response bias during the experiment. Two versions of the task were created to enable each correct and incorrect foil letter to be presented both above and below the mask and version was counterbalanced across participants.

Paired *t*-tests comparing psycholinguistic properties of the real word values across high and low imageability groups showed an expected significant difference in imageability [*t*(39) = –37.90, *p* < 0.001], but no differences in frequency, neighborhood size, bigram frequency, letter length, position of letter probed or neighbors at position probed [*t*(39) < 0.81, *p* > 0.42] (Appendix A of the Supplementary Material presents a table of word and nonword stimuli properties and Appendix B presents the results of a series of ANOVAs performed to ensure the properties of the real words were matched to each foil type).

#### Semantic Priming

Each participant was presented with 80 real words, 240 nonwords and 320 primes. The 80 real words were monosyllabic, 3–5 letter, low frequency English words. Due to the requirement to not overlap with the imageability set, the priming targets were drawn from the medium imageability range. Related primes for the 80 real word targets were selected using the Maki (2008) database. The real word targets were split into two lists and the related primes (e.g., *wood–LOG*) were shuffled to create unrelated items (e.g., *doll–LOG*). This resulted in two versions of the task, administration of which was counterbalanced across participants. The real words were again presented in two separate blocks of 40 according to prime status.

Again, three types of nonword items were created; pseudohomophones, pseudowords, and consonant strings. Eighty orthographically legal pseudohomophones were created and 80 prime words related to the pseudohomophone baseword were selected from the database. The pseudohomophones were split into two lists and the related primes (e.g., *key–LOK*) shuffled to create unrelated items (e.g., *beam-LOK*), which were again presented in two separate blocks. 80 orthographically legal pseudoword foils (e.g., *BOK*) and 80 orthographically illegal, unpronounceable consonant strings (e.g., *BPK)* were created in the same way as the imageability task and presented with the corresponding pseudohomophone primes. Again, the pseudowords and consonant strings were presented in two blocks of 40 items according to the pseudohomophone from which they were derived.

The correct letter and incorrect foil letters for each item were selected as in the imageability task and presented and counterbalanced in the same way. Paired *t*-tests revealed no significant differences across priming sets on imageability, which is desirable in that priming can be influenced by target imageability (Cortese et al., 1997). Nor were there any significant differences across priming sets on: frequency, neighborhood size, bigram frequency, letter length, position of the probed letter, neighbors at position probed, prime frequency, prime letter length, prime bigram frequency, prime and target forward association strength or prime and target semantic distance [*t*(39) < 1.90, *p* > 0.06] (Appendix C of the Supplementary Materials presents a table of word and nonword stimuli properties and Appendix D presents the results of a series of ANOVAs performed to ensure the properties of the real words were matched to each foil type).

### Procedure

Participants performed a two-choice letter detection task. Stimuli were presented using DMDX (Forster and Forster, 2003) and responses were made using a button box. All stimuli were presented in white uppercase 26-point font on a black background. For the imageability task, in each trial a fixation cross was presented in the center of the screen for 500 ms followed by a forward pattern mask “####” for 250 ms. A letter string was then presented for the length of time determined by the thresholding procedure before being replaced by a backward pattern mask. At a particular position, within this backward mask, a letter was presented above and a letter presented below and this remained visible for 4 s or until a response was made. For the semantic priming task, a prime word was presented in lowercase in the center of the screen for 500 ms immediately prior to presentation of the forward pattern mask.

When Reicher–Wheeler paradigms have determined exposure durations for each participant (as per Wheeler, 1970), rather than adopting a fixed brief duration (as per Reicher, 1969), this has been achieved using a staircase threshold procedure performed within a block of pre-experimental trials. This threshold procedure ascertains the level of exposure necessary to maintain accuracy at a predetermined level (generally 75%) and this specific duration is applied to each subsequent experimental trial in order to minimize ceiling and floor effects. Within the current paradigm experimental trials were blocked according to stimulus properties in order to maximize the probability of condition differences (Manelis, 1974). We therefore began each block with a 75% accuracy exposure duration determined in a pre-experimental block of a representative mixed stimulus composition, and from there applied a continuous adaptive staircase threshold procedure intended to persistently maintain 75% accuracy throughout each particular experimental block. This technique was intended to impose a constant perceptual demand upon the word processing system, thus minimizing habituation effects across the course of different blocks.

At the beginning of both the imageability and semantic priming tasks participants completed eight practice items with longer display durations before performing an initial thresholding block of 80 real word and nonword items presented in a pseudorandom order and created in the same way as the experimental stimuli. Within this thresholding block, the first item was presented for a duration of 250 milliseconds and this increased or decreased by 16.66 ms (one screen refresh rate) on every trial throughout the block in order to maintain as close to a 75% accuracy level as possible. At the end of the thresholding block, the final exposure duration was taken and applied as the starting duration for each subsequent experimental block for that individual participant. Following this initial block, participants performed the remaining 8 experimental blocks each containing 40 items. Within each new experimental block the initial exposure duration was set at the value derived from the initial thresholding block and this then increased and decreased in the same way throughout each experimental block to maintain 75% accuracy within that particular block. The order of presentation of the eight experimental blocks and of the 40 items within each block was random and was generated anew for each participant.

## Results

### Exposure Duration

Our use of a continuous adaptive staircase procedure meant that we could analyze the average exposure duration for each block as a dependent variable. Mean exposure duration provides a measure of the duration at which letters from a particular stimulus type could be detected on correctly on an average of 75% of occasions. We expected both lexical and semantic influences on average exposure durations.

#### Lexicality Effects

In order to investigate lexical influences in a manner comparable to previous studies of the (pseudo)word superiority effect, mean exposure duration was averaged across high and low imageability and related and unrelated blocks for each type of letter string. Repeated measure ANOVAs compared exposure duration across lexicality (words, pseudohomophones, pseudowords, consonant strings) by participants (*F*_1_) and items (*F*_2_) for both the imageability and priming task, with lexicality treated as a within-participant and between-item factor. Min*F*’ values (Clark, 1973) are also provided to indicate significance of effects over participants and items simultaneously.

**FIGURE 1:**
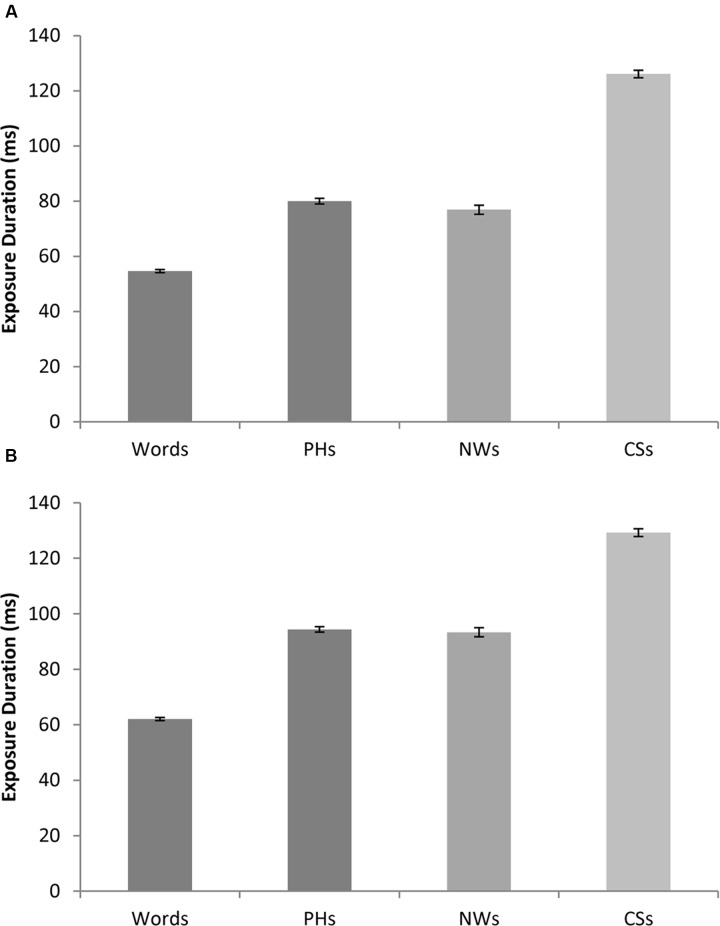
**(A)** Shows mean exposure durations for real words, pseudohomophones, pseudowords, and consonant strings, collapsed across the imageability manipulation. **(B)** Shows mean exposure durations for real words, pseudohomophones, pseudowords and consonant strings, collapsed across the semantic priming manipulation. Error bars are ± standard error (across items).

The ANOVA on the imageability stimuli revealed a significant main effect of lexicality [*F*_1_(3,189) = 49.77, *p* < 0.0005; *F*_2_(3,316) = 602.03, *p* < 0.0005; min*F*’(3,221) = 45.97, *p* < 0.0005]. As can be seen in **Figure 1A**, in line with the word superiority effect, real words required significantly shorter exposure durations than pseudohomophones [*F*_1_(1,63) = 29.16, *p* < 0.0005; *F*_2_(1,158) = 485.76, *p* < 0.0005; min*F*’(1,71) = 27.51, *p* < 0.0005], pseudowords [*F*_1_(1,63) = 39.44, *p* < 0.0005; *F*_2_(1,158) = 157.50, *p* < 0.0005; min*F*’(1,96) = 31.54, *p* < 0.0005], or consonant strings [*F*_1_(1,63) = 92.35, *p* < 0.0005; *t*_2_(158) = 2329.99, *p* < 0.0005; min*F*’(1,68) = 88.83, *p* < 0.0005]. In line with the pseudoword superiority effect, pseudoword legality was also found to influence exposure duration with consonant strings requiring significantly longer exposure durations than both pseudohomophones [*F*_1_(1,63) = 55.50, *p* < 0.0005; *F*_2_(1,158) = 745.29, *p* < 0.0005; min*F*’(1,73) = 51.65, *p* < 0.0005] and pseudowords [*F*_1_(1,63) = 32.60, *p* < 0.0005; *F*_2_(11,58) = 520.30, *p* < 0.0005; min*F*’(1,71) = 30.68, *p* < 0.0005]. The difference between exposure durations for pseudohomophones and pseudowords was not reliable by items [*F*_1_(1,63) = 6.00, *p* = 0.017; *F*_2_(1,158) = 2.53, *p* = 0.118; min*F*’(1,221) = 1.78, *p* = 0.184].

The ANOVA on the semantic priming stimuli revealed a significant main effect of lexicality [*F_1_*(3,189) = 22.62, *p* < 0.0005; *F*_2_(3,316) = 739.85, *p* < 0.0005; min*F*’(3,201) = 21.95, *p* < 0.0005]. As can be seen in **Figure 1B**, real words required significantly shorter exposure durations than pseudohomophones [*F*_1_(1,63) = 17.39, *p* < 0.0005; *F*_2_(1,158) = 635.54, *p* < 0.0005; min*F*’(1,66) = 16.93, *p* < 0.0005], pseudowords [*F*_1_(1,63) = 13.99, *p* < 0.0005; *F*_2_(1,158) = –6.10, *p* < 0.0005; min*F*’(1,221) = 4.25, *p* = 0.041] or consonant strings [*F*_1_(1,63) = 50.27, *p* < 0.0005; *F*_2_(1,158) = 1907.94, *p* < 0.0005; min*F*’(1,66) = 48.98, *p* < 0.0005]. Similarly, pseudoword legality was again found to influence exposure duration with consonant strings requiring significantly longer exposure durations than both pseudohomophones [*F*_1_(1,63) = 23.14, *p* < 0.0005; *F*_2_(1,158) = 474.37, *p* < 0.0005; min*F*’(1,69) = 22.06, *p* < 0.0005] and pseudowords [*F*_1_(1,63) = 16.40, *p* < 0.0005; *F*_2_(1,158) = 521.21, *p* < 0.0005; min*F*’(1,67) = 15.90, *p* < 0.0005]. No significant differences in exposure duration for pseudohomophones and pseudowords were found [*F*_1_(1,63) = 0.20, *p* = 0.656; *F*_2_(1,158) = 0.59, *p* = 0.443; min*F*’(1,108) = 0.15, *p* = 0.700].

#### Semantic Effects

As the semantic manipulation was relevant for only real words and pseudohomophones, analyses were restricted to these stimulus types and average exposure durations were compared across high and low imageability and related and unrelated priming blocks.

**FIGURE 2:**
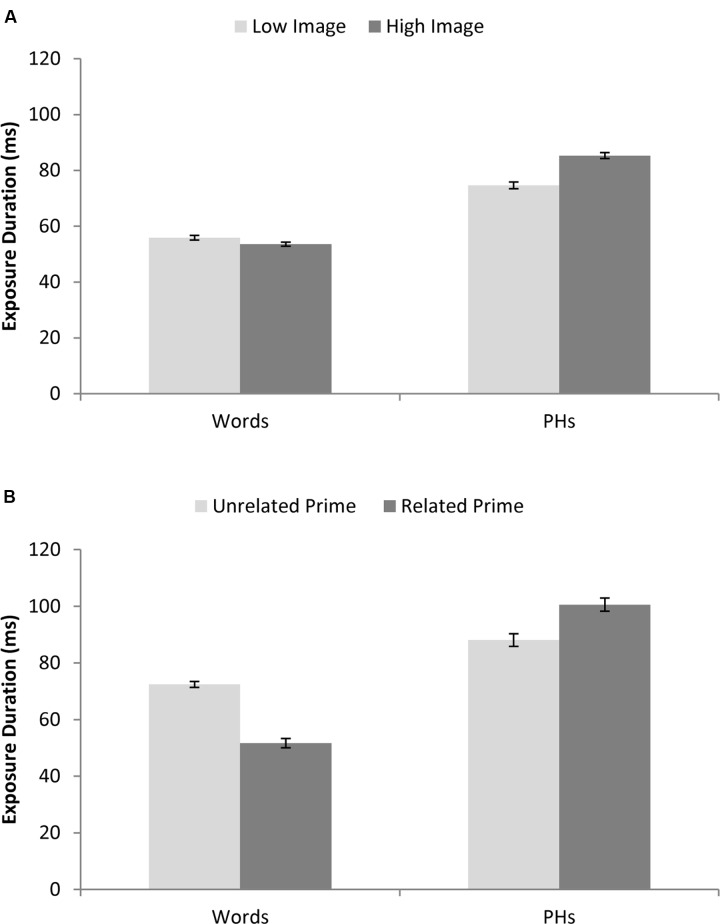
**(A)** Shows average exposure durations for real words and pseudohomophones varying in their imageability. **(B)** Shows average exposure durations for real words and pseudohomophones for preceded by a semantically related or unrelated prime. Error bars are ± standard error (across items).

Imageability was treated as a within-participant and between-item factor. There was a significant effect of imageability for real words by items but not participants [*F*_1_(1,63) = 0.31, *p* = 0.579; *F*_2_(1,78) = 4.45, *p* = 0.038; min*F*’(1,72) = 0.29, *p* = 0.592]. As shown in **Figure 2A** high imageability real words required shorter exposure durations than low imageability real words. A significant effect of imageability by items but not participants was also observed for the pseudohomophones [*F*_1_(1,63) = 2.37, *p* = 0.130; *F*_2_(1,78) = 44.36, *p* < 0.0005; min*F*’(1,70) = 2.25, *p* = 0.138]. As illustrated in **Figure 2A** high imageability pseudohomophones required *longer* exposure durations than low imageability pseudohomophones.

Semantic priming was treated as a within-participant and within-item factor. There was a significant effect of priming for real words [*F*_1_(1,63) = 6.40, *p* = 0.014; *F*_2_(1,79) = 89.11, *p* < 0.0005; min*F*’(1,72) = 5.97, *p* = 0.017]. As shown in **Figure 2B** real words preceded by a related prime required shorter exposure durations than words with an unrelated prime. A significant effect of semantic priming was also observed for the pseudohomophones by items but not participants [*F*_1_(1,63) = 2.47, *p* = 0.121; *F*_2_(1,79) = 9.12, *p* = 0.003; min*F*’(1,96) = 1.94, *p* = 0.167]. As illustrated in **Figure 2B** pseudohomophones preceded by a related prime required *longer* exposure durations than pseudohomophones with an unrelated prime.

### Reaction Time

In addition to examining how lexical and semantic variables affect average exposure durations needed to maintain 75% decision accuracy, we can also consider the influence of these factors on decision latency.

#### Lexicality Effects

In order to investigate lexical influences on reaction time, values across high and low imageability and related and unrelated blocks were averaged for each type of letter string. Repeated measure ANOVAs compared reaction time across lexicality (words, pseudohomophones, pseudowords, consonant strings) by participants (*F*_1_) and items (*F*_2_) for both the imageability and priming task, with lexicality treated as a within-participant and between-item factor.

**FIGURE 3:**
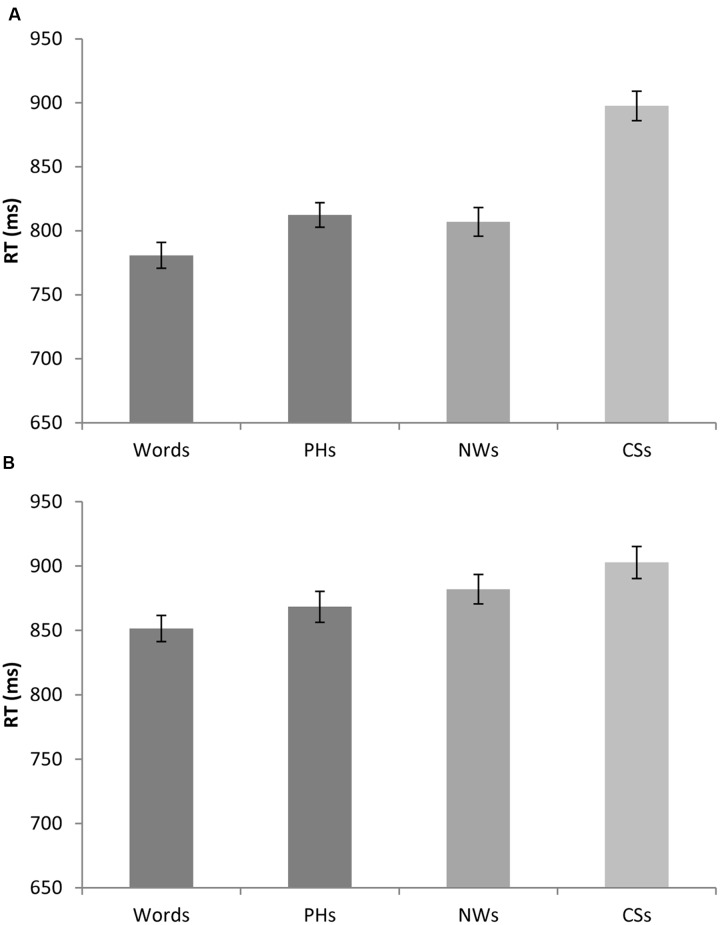
**(A)** Shows average reaction times for real words, pseudohomophones, pseudowords and consonant strings, collapsed across the imageability manipulation. **(B)** Shows average reaction times for real words, pseudohomophones, pseudowords and consonant strings, collapsed across the priming manipulation. Error bars are ± standard error (across items).

The ANOVA on the imagaeability stimuli revealed a significant main effect of lexicality [*F*_1_(3,189) = 8.61, *p* < 0.0005; *F*_2_(3,316) = 7.39, *p* < .0005 min*F*’(3,489) = 3.98, *p* = 0.008]. As can be seen in **Figure 3A**, *t*-tests revealed that real words were processed similarly to pseudohomophones [*F*_1_(1,63) = 0.49, *p* = 0.490; *F*_2_(1,158) = 0.25, *p* = 0.617; min*F*’(1,218) = 0.17, *p* = 0.685] and pseudowords [*F*_1_(1,63) = 0.61, *p* = 0.436; *F*_2_(1,158) = 0.87, *p* = 0.355; min*F*’(1,152) = 0.36, *p* = 0.550], but more rapidly than consonant strings [*F*_1_(1,63) = 31.03, *p* < 0.0005; *F*_2_(1,158) = 14.14, *p* < 0.0005; min*F*’(1,489) = 9.71, *p* = 0.002]. Pseudoword legality was also found to influence reaction times with consonant strings requiring significantly longer exposure durations than both pseudohomophones [*F*_1_(1,63) = 16.08, *p* < 0.0005; *F*_2_(1,158) = 18.66, *p* < 0.0005; min*F*’(1,168) = 8.64, *p* = 0.003] and pseudowords [*F*_1_(1,63) = 14.67, *p* < 0.0005; *F*_2_(1,158) = 10.38, *p* = 0.008; min*F*’(1,204) = 6.08, *p* = 0.015]. The difference between reaction times to pseudohomophones and pseudowords was not reliable [*F*_1_(1,63) = 1.90, *p* = 0.171; *F*_2_(1,128) = 2.02, *p* = 0.157; min*F*’(1,175) = 0.98, *p* = 0.324].

The ANOVA on the semantic priming stimuli revealed a significant main effect of lexicality [*F*_1_(3,189) = 4.08, *p* = 0.008; *F*_2_(3,316) = 3.54, *p* = 0.015 min*F*’(3,488) = 1.90, *p* = 0.130]. As can be seen in **Figure 3B**, real words were processed similarly to pseudohomophones [*F*_1_(1,63) = 0.46, *p* = 0.402; *F*_2_(1,158) = 1.15, *p* = 0.286; min*F*’(1,116) = 0.33, *p* = 0.568], but somewhat more efficiently than pseudowords [*F*_1_(1,63) = 3.53, *p* = 0.065; *F*_2_(1,158) = 3.96, *p* = 0.048; min*F*’(1,171) = 1.87, *p* = 0.173] and consonant strings [*F*_1_(1,63) = 12.11, *p* < 0.0005; *F*_2_(1,158) = 10.18, *p* = 0.002; min*F*’(1,193) = 5.53, *p* = 0.020]. Similarly, pseudoword legality influenced RTs with consonant strings requiring significantly longer exposure durations than pseudohomophones [*F*_1_(1,63) = 7.34, *p* = 0.009; *F*_2_(1,158) = –4.00, *p* = 0.048; min*F*’(1,216 = 2.59, *p* = 0.109] but not pseudowords [*F*_1_(1,63) = 1.06, *p* = 0.309; *F*_2_(1,158) = 1.51, *p* = 0.219; min*F*’(1,153) = 0.62, *p* = 0.431]. No significant differences in processing efficiency for pseudohomophones and pseudowords were found [*F*_1_(1,63) = 1.88, *p* = 0.176; *F*_2_(1,158) = 0.67, *p* = 0.412; min*F*’(1,220) = 0.49, *p* = 0.517].

#### Semantic Effects

Analyses were of semantic effects were again restricted to words and pseudohomophones and average reaction time was compared across high and low imageability and related and unrelated priming blocks.

**FIGURE 4:**
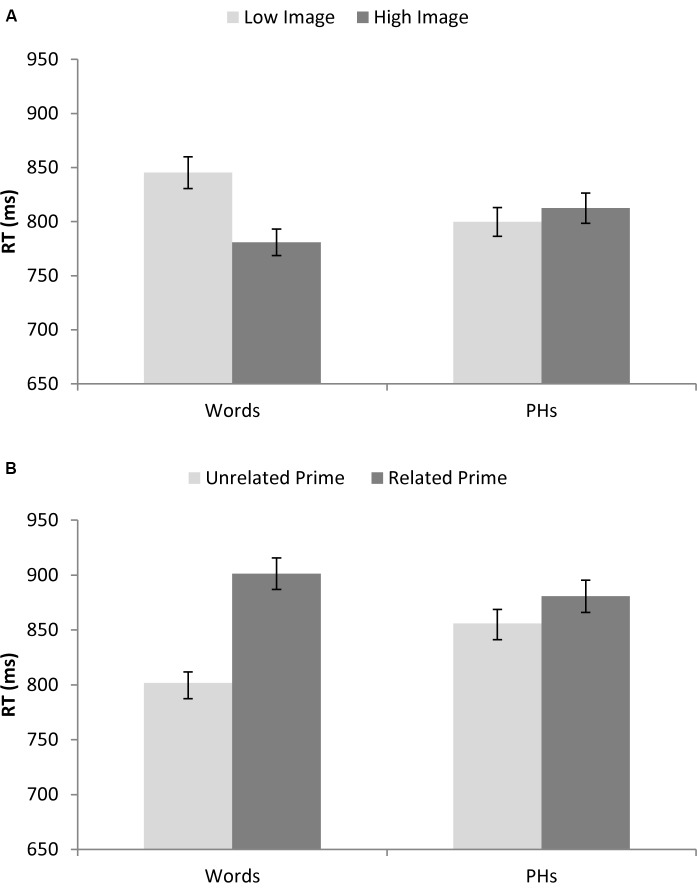
**(A)** Shows average reaction times for real words and pseudohomophones varying in their imageability. **(B)** Shows average reaction times for real words and pseudohomophones for preceded by a related or unrelated semantic prime. Error bars are ± standard error (across items).

Imageability was treated as a within-participant and between item factor. There was a significant effect of imageability for real words, as seen for average exposure duration. As depicted in **Figure 4A** decisions about letters in high imageability real words were significantly faster than low imageability real words [*F*_1_(1,63) = 5.11, *p* = 0.027; *F*_2_(1,78) = 11.42, *p* = 0.001; min*F*’(1,114) = 3.53, *p* = 0.063] whilst no significant effect of imageability was found for the pseudohomophones [*F*_1_(1,63) = 0.12, *p* = 0.731; *F*_2_(1,78) = 0.44, *p* = 0.511; min*F*’(1,96) = 0.09, *p* = 0.760].

Semantic priming was treated as a within-participant and within-item factor. There was a significant *inhibitory* effect of semantic priming for real words, in contrast to the effect observed for average exposure duration. As depicted in **Figure 4B**, decisions about letters in real words preceded by a related prime related were *slower* than an unrelated prime [*F*_1_(1,63) = 14.29, *p* < 0.0005; *F*_2_(1,79) = 49.42, *p* < 0.0005; min*F*’(1,98) = 11.09, *p* = 0.001] whilst no significant effect of priming was found for the pseudohomophones [*F*_1_(1,63) = 0.98, *p* = 0.326; *F*_2_(1,79) = 3.31, *p* = 0.072; min*F*’(1,99) = 0.76, *p* = 0.387].

## Discussion

This study set out to examine whether both lexical and semantic information can influence letter identification performance. We used blocked stimulus presentation and a continuous adaptive staircase procedure meaning that we could examine average exposure durations for each item for a given level of accuracy (75%), and also reaction times for forced choice decisions about letter identity. In terms of lexical influences, clear word and pseudoword superiority effects were obtained in average exposure duration both the imageability and priming stimuli: participants required significantly shorter exposure to real words than pseudowords and to pseudowords than consonant strings in order to accurately identify constituent letters. Numerically, a similar pattern was obtained in reaction times. The data therefore provide robust evidence for lexical influence on letter identification, particularly in the perceptual measure of exposure duration. This aligns with accuracy differences in past research that have used mixed presentation of stimulus types with fixed exposure durations (Reicher, 1969; Wheeler, 1970), confirming the comparability of our adaptive thresholding paradigm.

Interestingly, we found no evidence of any advantage for letter identification in pseudohomophones relative to pseudowords, and hence we failed to observe the pseudohomophone superiority effect (Chastain, 1987). In fact, if anything, there was a numerical disadvantage for pseudohomophones relative to nonwords in both exposure duration and also in reaction times for the imageability stimuli. Although this could be interpreted as arguing against a role for top–down influence of phonology, the pseudohomophone superiority effect is likely to be critically influenced by the type of letter distractors used. In Chastain’s original report, the alternative letter always made another pseudohomophone (e.g., PAED probed with a P or an R). Unfortunately this was not possible to achieve with the current stimuli, and we chose to use an alternative letter that formed another real word, as this removed lexical phonology as a basis for letter identification. The consequence of doing this is that any benefit of phonological familiarity for pseudohomophones is offset by a response bias toward a letter than makes a familiar word. Indeed, when the alternative letter is contained within the pseudohomophone’s baseword, a significant disadvantage for pseudohomophones relative to pseudowords has been reported (Hooper and Paap, 1997).

Turning to the key novel manipulations of our study, we considered the extent to which top–down semantic information influences letter identification by manipulating the intra-word dimension of imageability and the inter-word dimension of semantic priming. We observed lower exposure durations were needed for high relative to low imageability words, and although this effect was not significant across participants, the same effect was significant over both participants and items in reaction times. The facilitative effect of imageability mirrors that found in our previous lexical decision study using the same stimuli (Evans et al., 2012), and can be attributed to the activation of more robust semantic representations for high relative to low imageability words (Plaut and Shallice, 1993; Harm and Seidenberg, 2004). It is possible that greater top–down activation from semantics for high than low imageability words took time to build, producing a limited effect on exposure duration and a stronger effect on decision latency. Overall then, our study provides the first evidence that letter identification is influenced by the semantic dimension of imageability.

It is notable that we also observed a reverse imageability effect in exposure duration for pseudohomopohnes, such that longer exposure durations were needed for items with high relative to low imageability basewords, an effect that was again not significant across participants and, in contrast to words, was not apparent in reaction times. Exposure durations were longer for pseudohomophones than words, presumably because of a need to avoid inaccurate perception of the baseword. High imageability pseudohomophones would receive more top–down activation of the letters of the baseword than low imageability pseudohomophones, meaning extra exposure duration would be needed. However, at the later point at which a decision is being made, the bottom-up activation of the letters of the pseudohomophone would have overcome activation of the baseword, meaning no effect is observed in reaction times.

Turning to the semantic priming manipulation, we observed lower exposure durations for words preceded by semantically related than unrelated primes and this difference was reliably significant. This is likely because in the priming paradigm the additional semantic information in related trials occurs before the onset of the critical stimulus, the impact of the top–down semantic activation for related targets is apparent earlier than in the case of imageability. This semantic priming effect on exposure duration parallels the facilitative effect observed in lexical decision using the same stimuli (Evans et al., 2012), and is in line with the view that presentation of a related prime speeds access to the meaning of the target by activating shared semantic features (Plaut and Booth, 2000). This study therefore represents the first evidence that letter identification is influenced by the dimension of semantic priming.

We also observed a reverse semantic priming effect for pseudohomophones in exposure durations by items but not participants, but no effect on reaction times. This is a very similar pattern to that seen for the reversed imageability effect observed for pseudohomophones, and can be explained in the same way. Pre-activation of sematic representations of the baseword by a related prime would mean greater top–down activation of the baseword, relative to the case of an unrelated prime. Extra exposure would therefore be needed to prevent semantically primed relative to unprimed pseudohomophones being mistaken for their basewords, producing the observed reversal in the semantic priming effect.

One unexpected finding from the semantic priming manipulation was the observation of a large and highly significant inhibitory semantic priming effect on decision latency, such that reaction times to words preceded by a related prime were much slower than an unrelated prime. This result does not just run counter to the exposure duration effect reported here, but also the facilitative effect typically observed in other word processing tasks, such as lexical decision (Neely, 1991), including in our previous study using the same items (Evans et al., 2012). As such, it is worth considering whether this finding may in fact be an artifact of some aspect of our methodology. Firstly, we used a continuous adaptive staircase procedure as we were interested in exposure duration as a dependent variable. Yet we observed consistent and robust word and pseudoword superiority effects comparable to those seen when fixed exposure durations have been used and accuracy of identification examined. Secondly, we blocked our stimuli by condition, again because we were interested in exposure duration as a measure. It is known from work in lexical decision and naming that blocking does impact on a number of lexical effects, such as frequency (Lupker et al., 1997; Kinoshita and Mozer, 2006), with stimulus mixing decreasing effect size. Hence while the blocked presentation used here may have enhanced the effects observed, there is no reason to think that it would reverse their direction, as seen for semantic priming. Lastly, it is worth noting that the reversal observed in RT relative to exposure duration was specific to semantic priming, with effects running in the same direction across both measures for the imageability manipulation and also the word and nonword superiority effects. Hence it does not seem likely that slower responses to stimuli presented with a related prime were purely the result of the shorter exposure duration needed to achieve the requisite level of accuracy. Hence, the reversed semantic priming effect we observed does seem to be a genuine effect, albeit a task and measure specific one.

If one considers the consequences of semantic feedback for an inter-word semantic variable such as semantic priming, the reversed effect observed in reaction time may be explained. Semantic priming is assumed to exert an effect through a related prime heightening the activation of the meaning of the subsequent target; hence producing a lexical decision advantage for that target, and the exposure duration advantage seen in the current study. However, this assumption posits that the activated target should also maintain activation of the representation of the previously presented related prime. Within a fully interactive word recognition network, both the related prime and target would thus feedback to influence the letter level, increasing letter-level competition and producing interference for letter detection tasks. In contrast, a semantically unrelated prime and target would not maintain this reciprocal activation; hence the unrelated prime is less likely to feedback to the letter level to produce competing activation. If we assume that the target’s activation of the related prime builds over time, this would explain why the reversal was only apparent in RT.

Some support for this prime feedback interference account is provided by the fact that RTs in general were longer for the semantic priming stimuli than the imageability stimuli. This contrasts to results seen for the same stimuli when presented in the context of a visual lexical decision task (Evans et al., 2012), where if anything it was the imageability stimuli that proved slower than the semantic priming stimuli. This general slowing of letter identification decisions for the semantic priming stimuli relative to the imageability stimuli suggests an overall interference effect from the prior presentation of the prime on forced choice letter identification decisions. The proposal being made here to account for the reverse priming effect seen in reaction times is that this this general interference effect is significantly enhanced when the prime is semantically related to the target, as this boosts the activation of letters in the prime, which delays decision relative to trials where the target is unrelated.

A similar interference effect may contribute to the reversal of the standard pseudoword and pseudohomophone advantages that have been reported in the literature for accuracy when using fixed exposure durations and mixed presentation. As noted earlier, Hooper and Paap (1997) found that letter perception in pseudohomophones was worse than in pseudowords when the distractor letter was in the pseudohomophone’s baseword (e.g., *PURT probed with U and E*). As shown by the reversed semantic effects observed in the present study, perception of the pseudohomophone strongly activates its baseword due to its shared phonology, which then flows back to its component letters, which both promotes the distractor but also interferes with activation of the target letter. A parallel result was obtained by Grainger and Jacobs (2005) using pseudowords derived from “hermit” words, with no orthographic neighbors. When probed with a distractor letter that was in the orthographic neighbor (e.g., *UPLY probed with P and G*) performance became worse than when the target letter was presented in a row of Xs. In this case, there is strong activation of the single orthographic neighbor as there would be little inhibition from other orthographically similar items, which then flows back to its component letters, promoting selection of the distractor and interfering with activation of the target letter. These disadvantages seem comparable to the reversed semantic priming effect here in that strong activation of the representations of competing words interferes with activation and selection of the target letter.

Across the intra-word dimension of imageability and the inter-word dimension of semantic priming, our results provide the first evidence of feedback from the semantic level to the letter level that parallels the lexical influences previously reported in this task (Reicher, 1969; Wheeler, 1970; Rumelhart and McClelland, 1982). The facilitative effect of imageability is considered to stem from highly imageable words possessing ‘richer’ semantic representations, which are activated over a larger set of more consistently accessed semantic features (Plaut and Shallice, 1993; Harm and Seidenberg, 2004). Alternative interpretations of this effect in lexical decision exist depending on where one considers the locus of lexical decision to be (Borowsky and Besner, 1993; Plaut, 1997). Yet many accounts assume that imageability effects are a product of feedback from the semantic level to the lexical level in localist models (Coltheart et al., 2001) or the orthographic level in distributed models (Van Orden et al., 1990; Pexman and Lupker, 1999; Harm and Seidenberg, 2004; cf. Chang et al., 2013), consistent with the effect we have observed in letter detection.

The semantic priming advantage seen in lexical decision can similarly be attributed to semantic feedback. Within localist models within-level semantic or between-level semantic/lexical spreading activations produce increased activation of the related target (Collins and Loftus, 1975; Stolz and Besner, 1997), whilst distributed models assume that shared patterns of activation across the semantic units enable accelerated activation of related targets (Plaut, 1995; Plaut and Booth, 2000). Such accounts are compatible with the facilitative effect of semantic priming we observed in exposure duration. Although we obtained an inhibitory effect of semantic priming in reaction times, this could be attributed to interference at the decision stage from the letters in the prime adding noise to the decision, and this interference being stronger for related than unrelated trials as a consequence of stronger top–down activation of prime letters. Hence, semantic feedback to letter-level processing provides a plausible account for the patterns of both imageability and semantic priming effects we observed across exposure durations and reaction times, but it remains to be seen whether this pattern can be simulated within interactive models of orthographic processing.

The current demonstration that both lexical and semantic information can provide top–down influences upon letter processing provides a future simulation target for models of visual word recognition. Both localist and connectionist models suggest that semantic information can influence letter processing via feedback, within localist models this is mediated by the orthographic lexicon whilst in connectionist models this influence is more direct (Coltheart et al., 2001; Harm and Seidenberg, 2004). A failure to detect semantic influences upon letter processing in the presence of lexical effects could be readily accounted for within localist models. Distributed accounts, however, would seem to predict that lexical-level effects should be accompanied by semantic influences. Although the current findings cannot discriminate between these two approaches, the demonstration of significant semantic influences upon letter processing provides supportive evidence for the more central role of semantic information within connectionist models.

These results thus provide behavioral evidence for the existence of a high degree of rapid interactivity within the word recognition system. This interactivity has also been detected at the neural level, with a growing body of imaging evidence demonstrating the top–down influence of higher-semantic processing on lower perceptual areas (Hon et al., 2009; Liu et al., 2010; Tworney et al., 2011). Similarly, electrophysiological evidence also suggests that semantic processing initiates very rapidly during the course of word recognition (Pulvermuller et al., 2001; Hauk et al., 2006; Sysoeva et al., 2007; Wirth et al., 2008; Fujimaki et al., 2009). Although the neural mechanisms underlying semantic feedback remain undetermined, it is possible that recurrent feedback connections enable rapidly activated semantic information to play a functional role within even the earliest stages of word processing where the identity of component letters is determined. The extent of perceptual processing required to activate these higher-order representations remains a matter for further investigation, however, the current results indicate that rather than passively perceiving input, higher cognitive mechanisms proactively attempt to understand the environmental input (Bar, 2009). This ability may function to increase the efficiency of the network (Bar, 2003), a mechanism that would prove particularly beneficial to letter processing given the perceptual demands posed by the requirement to detect minor variations in alphabetic stimuli given large variations in case, font and size.

In summary, our results provide the first demonstration of semantic influences on letter identification. This finding is highly compatible with previous lexical decision studies that report significant effects of imageability, semantic priming and other meaning-level variables. Our research extends this work by considering exposure duration to demonstrate that semantic information can influence the processes of letter identification that supports accurate word recognition. The meaning-level influences we have observed suggest that visual word recognition is underpinned by a fully interactive network in which even the earliest stages of orthographic processing involve activation of meaning.

## Ethics Statement

This study was approved by University of Manchester School of Psychological Sciences Research Ethics Committee. Informed consent was obtained from all participants who had previously had more than 24 h to consider the Participant Information Sheet before attending the experiment, and who were again presented with this Sheet and asked if they had any questions before the consent form was presented.

## Author Contributions

GE designed the research, collected the data, analyzed the data, and wrote the paper. ML designed the research and wrote the paper. AW designed the research, analyzed the data, and wrote the paper.

## Conflict of Interest Statement

The authors declare that the research was conducted in the absence of any commercial or financial relationships that could be construed as a potential conflict of interest.
