# Supplementary material for: Seeing the Meaning: Top–Down Effects on Letter Identification
Source: Front Psychol. 2017 Apr 20;8:322. doi: 10.3389/fpsyg.2017.00322 (PMC5397417; doi:10.3389/fpsyg.2017.00322)
Supplement: Supplementary file 1 [file Data_Sheet_1.pdf]

Online Supplemental Materials

Appendix A

Means (and standard deviations) of a range psycholinguistic variables for each imageability set and the three nonword foil types.

Variable	Real words		Nonword Foils		
	High imageability	Low imageability	Pseudo-homophones	Pseudo-words	Consonant strings
Imageability[ac]	6.11(0.47)	2.72(0.41)	4.54(1.70)	-	-
Celex frequency[bc]	12.81(10.46)	10.67(9.21)	12.87(12.92)	-	-
Neighbourhood size[b]	9.18(4.59)	9.18(4.59)	7.46(4.65)	7.59(4.65)	0.59(1.07)
Bigram frequency[b]	1670.41 (1018.59)	1452.53 (1310.87)	1259.60 (1022.69)	1319.93 (1070.48)	266.53 (501.99)
Letter length	4.00(0.72)	4.00(0.72)	4.00(0.72)	4.00(0.72)	4.00(0.72)
Position of probed letter	2.48(1.20)	2.52(1.20)	2.57(1.29)	2.39(1.17)	2.41(1.18)
Neighbours at position probed[b]	3.05(1.81)	3.12(1.86)	3.45(2.46)	2.75(3.07)	0.19(0.63)

[a]*Values taken from Cortese and Fugett (2004) database,* [b]*Values taken from N-watch (Davis, 2005),* [c]*Values taken for pseudohomophone baseword.*

Appendix B

Statistical comparisons of stimulus properties in the imageability task. Results of 2(imageability: high, low) by 2(lexicality: real word, foil type) factorial ANOVAs comparing real words to each foil type on a variety of psycholinguistic variables. The pseudowords and consonant strings were coded for the imageability of the pseudohomophone foil from which they were derived.

Variable	*Imageability*	*Lexicality*	*Imageability × Lexicality*
Real words and Pseudohomophones:			
Imageability[ac]	F(1,74)=1664.59, p<0.001	F(1,74)=0.14,p=0.71	F(1,74)=2.47,p=0.12
Celex frequency[bc]	F(1,78)=0.39,p=0.53	F(1,78)=0.32,p=0.57	F(1,78)=0.56,p=0.46
Neighbourhood size[b]	F(1,78)=0.70,p=0.40	F(1,78)=3.03,p=0.09	F(1,78)=0.70,p=0.40
Bigram frequency[b]	F(1,78)=0.58,p=0.45	F(1,78)=3.17,p=0.08	F(1,78)=0.27,p=0.60
Letter length	F(1,78)<0.01,p>0.99	F(1,78)<0.01,p>0.99	F(1,78)<0.01,p>0.99
Position of probed letter	F(1,78)=0.31,p=0.86	F(1,78)=0.95, p=0.76	F(1,78)=0.28,p=0.60
Number of neighbours at position of letter probed[b]	F(1,78)=0.01,p=0.96	F(1,78)=0.82,p=0.37	F(1,78)=0.10,p=0.75
Real words and Pseudowords:			
Neighbourhood size[b]	F(1,78)=2.45,p=0.12	F(1,78)=2.85,p=0.10	F(1,78)=2.45,p=0.12
Bigram frequency[b]	F(1,78)=0.28,p=0.60	F(1,78)=1.67,p=0.20	F(1,78)=0.54,p=0.46
Letter length	F(1,78)<0.01,p>0.99	F(1,78)<0.01,p>0.99	F(1,78)<0.01,p>0.99
Position of probed letter	F(1,78)=0.49,p=0.48	F(1,78)=0.20,p=0.66	F(1,78)=1.59,p=0.21
Number of neighbours at position of letter probed[b]	F(1,78)=0.06,p=0.80	F(1,78)=0.57,p=0.45	F(1,78)=0.22,p=0.64
Real words and Consonant Strings:			
Neighbourhood size[b]	F(1,78)=0.25,p=0.62	F(1,78)=133.04,p<0.001	F(1,78)=0.25,p=0.62
Bigram frequency[b]	F(1,78)=0.13,p=0.72	F(1,78)=80.05,p<0.001	F(1,78)=1.33,p=0.25
Letter length	F(1,78)<0.01,p>0.99	F(1,78)<0.01,p>0.99	F(1,78)<0.01,p>0.99
Position of probed letter	F(1,78)=0.02,p=0.87	F(1,78)=0.12,p=0.73	F(1,78)=0.63,p=0.43
Number of neighbours at position of letter probed[b]	F(1,78)=1.72, p=0.19	F(1,78)=98.80,p<0.001	F(1,78)=0.28,p=0.60

[a]Values taken from Cortese and Fugett (2004) database, [b]Values taken from N-watch (Davis, 2005), [c]Values taken for pseudohomophone baseword

Appendix C

Means (and standard deviations) of a range psycholinguistic variables for each priming set and the three nonword foil types.

Variable	Real Words		Nonword Foils		
	Set 1	Set 2	Pseudo-homophones	Pseudo-words	Consonant strings
Target:					
Imageability[ad]	4.96(1.17)	4.95(1.18)	5.20(1.21)	-	-
Celex frequency[bd]	13.08(8.12)	13.87(12.89)	17.84(17.05)	-	-
Neighbourhood size[b]	9.75(4.31)	9.75(4.31)	8.36(3.45)	9.45(4.38)	0.59(1.00)
Bigram frequency[b]	1587.70 (1237.24)	1591.46 (1353.35)	1362.05 (1145.12)	1342.78 (1060.45)	389.32 (1131.76)
Letter length	4.00(0.72)	4.00(0.72)	4.00(0.72)	4.00(0.72)	4.00(0.72)
Position of probed letter	2.35(1.25)	2.35(1.25)	2.63(1.17)	2.45(1.21)	2.34(1.23)
Neighbours at position probed[b]	3.37(2.34)	3.25(2.22)	3.29(2.48)	3.39(3.28)	0.14(0.58)
Prime:					
Celex frequency[b]	38.15 (40.67)	37.89 (69.77)	61.24 (144.52)	61.24 (144.52)	61.24 (144.52)
Letter length[b]	5.28(1.45)	5.03(1.25)	4.28(1.26)	4.28(1.26)	4.28(1.26)
Bigram frequency[b]	1163.27 (1101.74)	1438.56 (1502.76)	1408.89 (1315.70)	1408.89 (1315.70)	1408.89 (1315.70)
Related prime and target:					
Forward association strength[cd]	0.10(0.14)	0.09(0.12)	0.09(0.16)	-	-
Semantic distance[cd]	8.95(7.67)	7.94(7.72)	8.59(6.55)	-	-

[a]Values taken from Cortese and Fugett (2004) database, [b]Values taken from N-watch (Davis, 2005), [c]Values taken from Maki (2008) database, [d]Values taken for pseudohomophone baseword.

Appendix D

Statistical comparisons of stimulus properties in the semantic priming task. Results of 2(priming: related, unrelated) by 2(lexicality: real word, foil type) factorial ANOVAs comparing real words to each foil type on a variety of psycholinguistic variables. The pseudowords and consonant strings were coded for the relatedness of the pseudohomophone foil from which they were derived.

Variable	*Priming set*	*Lexicality*	*Set × Lexicality*
Real words and Pseudohomophones:			
Imageability[ac]	F(1,76)=0.29,p=0.60	F(1,76)=2.14,p=0.15	F(1,76)=0.39,p=0.53
Celex frequency[bc]	F(1,78)=0.03,p=0.87	F(1,78)=3.39,p=0.07	F(1,78)=0.04,p=0.84
Neighbourhood size[b]	F(1,78)=0.24,p=0.62	F(1,78)=2.93,p=0.09	F(1,78)=0.24,p=0.62
Bigram frequency[b]	F(1,78)=0.10,p=0.75	F(1,78)=1.30,p=0.26	F(1,78)=0.11,p=0.74
Letter length	F(1,78)<0.01,p>0.99	F(1,78)<0.01,p>0.99	F(1,78)<0.01,p>0.99
Forward association strength[cd]	F(1,78)=0.20,p=0.65	F(1,78)=0.01,p=0.91	F(1,78)=0.05,p=0.83
Semantic distance[cd]	F(1,78)=0.64,p=0.43	F(1,78)=0.02,p=0.90	F(1,78)=0.01,p=0.94
Position of probed letter	F(1,78)=0.22,p=0.64	F(1,78)=1.20, p=0.27	F(1,78)=0.22,p=0.64
Number of neighbours at position of letter probed[b]	F(1,78)=0.86,p=0.36	F(1,78)=0.01,p=0.96	F(1,78)=2.08,p=0.15
Real words and Pseudowords:			
Neighbourhood size[b]	F(1,78)<0.01,p>0.99	F(1,78)=0.11,p=0.74	F(1,78)<0.01,p>0.99
Bigram frequency[b]	F(1,78)=0.23,p=0.63	F(1,78)=1.85,p=0.18	F(1,78)=0.25,p=0.62
Letter length	F(1,78)<0.01,p>0.99	F(1,78)<0.01,p>0.99	F(1,78)<0.01,p>0.99
Position of probed letter	F(1,78)<0.01,p>0.99	F(1,78)=0.14,p=0.70	F(1,78)<0.01,p>0.99
Number of neighbours at position of letter probed[b]	F(1,78)=3.06,p=0.09	F(1,78)=0.02,p=0.89	F(1,78)=1.66,p=0.20
Real words and Consonant Strings:			
Neighbourhood size[b]	F(1,78)=0.01,p=0.91	F(1,78)=175.33,p<0.001	F(1,78)=0.01, p=0.91
Bigram frequency[b]	F(1,78)=0.53,p=0.47	F(1,78)=32.63,p<0.001	F(1,78)=0.49,p=0.48
Letter length	F(1,78)<0.01,p>0.99	F(1,78)<0.01,p>0.99	F(1,78)<0.01,p>0.99
Position of probed letter	F(1,78)=0.13,p=0.26	F(1,78)=0.00,p=0.96	F(1,78)=1.29,p=0.26
Number of neighbours at position of letter probed[b]	F(1,78)=0.11,p=0.74	F(1,78)=75.48,p<0.001	F(1,78)=4.11,p=0.05
Prime values Real words and all Foils:			
Celex frequency[b]	F(1,78)=0.06,p=0.80	F(1,78)=1.81,p=0.18	F(1,78)=0.07,p=0.79
Letter length[b]	F(1,78)=0.37,p=0.55	F(1,78)=0.70,p=0.40	F(1,78)=0.37,p=0.55
Bigram frequency[b]	F(1,78)=0.62,p=0.43	F(1,78)=0.28,p=0.60	F(1,78)=0.25,p=0.62

[a]Values taken from Cortese and Fugett (2004) database, [b]Values taken from N-watch (Davis, 2005), [c]Values taken from Maki (2008) database, [d]Values taken for pseudohomophone baseword

References

Cortese, M., & Fugett, A. (2004). Imageability ratings for 3,000 monosyllabic words. *Behav. Res. Methods Instrum. Comput. 36*, 384–387. doi: 10.3758/ BF03195585.

Davis, C. J (2005). N-Watch: A program for deriving neighbourhood size and other psycholinguistic statistics. *Behaviour Research Methods*, *37*, 65-70.

Maki, W. (2008). A database of associative strengths from the strength-sampling model: a theory based supplement to the Nelson, McEvoy and Schreiber word association norms. *Behav. Res. Methods 40*, 232–235.
